# Immunological Heterogeneity of Healthy Peripheral Blood Stem Cell Donors—Effects of Granulocyte Colony-Stimulating Factor on Inflammatory Responses

**DOI:** 10.3390/ijms19102886

**Published:** 2018-09-22

**Authors:** Tor Henrik Anderson Tvedt, Guro K. Melve, Galina Tsykunova, Aymen Bushra Ahmed, Annette K. Brenner, Øystein Bruserud

**Affiliations:** 1 Department of Medicine, Section for Hematology, Haukeland University Hospital, 5021 Bergen, Norway; glts@helse-bergen.no (G.T.); abah@helse-bergen.no (A.B.A.); Annette.Brenner@uib.no (A.K.B.); brus@helse-bergen.no (Ø.B.); 2 Institute of Clinical Science, Section for Hematology, University of Bergen, 5021 Bergen, Norway; guro.kristin.melve@helse-bergen.no; 3 Department of Immunology and Transfusion Medicine, Haukeland University Hospital, 5021 Bergen, Norway

**Keywords:** toll-like receptors, Interleukin-6, C-reactive protein, acute-phase reaction, graft versus host disease, tissue and organ procurement

## Abstract

Interleukin-6 (IL-6) contributes to the development of immune-mediated complications after allogeneic stem cell transplantation. However, systemic IL-6 levels also increase during granulocyte colony-stimulating factor (G-CSF) mobilization of hematopoietic stem cells in healthy donors, but it is not known whether this mobilization alters systemic levels of other IL-6 family cytokines/receptors and whether such effects differ between donors. We examined how G-CSF administration influenced C-reactive protein (CRP) levels (85 donors) and serum levels of IL-6 family cytokines/receptors (20 donors). G-CSF increased CRP levels especially in elderly donors with high pretherapy levels, but these preharvesting levels did not influence clinical outcomes (nonrelapse mortality, graft versus host disease). The increased IL-6 levels during G-CSF therapy normalized within 24 h after treatment. G-CSF administration did not alter serum levels of other IL-6-familly mediators. Oncostatin M, but not IL-6, showed a significant correlation with CRP levels during G-CSF therapy. Clustering analysis of mediator levels during G-CSF administration identified two donor subsets mainly characterized by high oncostatin M and IL-6 levels, respectively. Finally, G-CSF could increase IL-6 release by in vitro cultured monocytes, fibroblasts, and mesenchymal stem cells. In summary, G-CSF seems to induce an acute phase reaction with increased systemic IL-6 levels in healthy stem cell donors.

## 1. Introduction

Granulocyte colony-stimulating factor (G-CSF)-mobilized peripheral blood stem cell grafts are used for allogeneic stem cell transplantation (ALLO-SCT) [1]. This G-CSF therapy has several immediate effects on the donor immune system but does not seem to have any long-term consequences [2]. It increases levels of various anti-inflammatory cytokines while simultaneously decreasing the production of several proinflammatory cytokines [3,4], inhibits T cell responsiveness and shifts their differentiation towards Th2 responses [5,6], induces IL-10 producing allo-inhibitory regulatory T cells [7], promotes the development of myeloid-derived tolerogenic dendritic cells [8], and reduces serum levels of the chemotactic C-X-C motif ligand 8 (CXCL8) and C-X-C motif ligand 12 (CXCL12) chemokines [9]. Thus, anti-inflammatory effects are common [8]. 

The increased risk of graft-versus-host disease (GVHD) for patients receiving G-CSF-mobilized stem cells has been explained by the increased number of donor T cells in these grafts [10]. However, the effects of G-CSF therapy in healthy individuals are complex as illustrated both by the frequent reversible side effects (e.g., musculoskeletal pain) and uncommon but more severe toxicity (e.g., splenic rupture and pulmonary toxicity), including progression of arthritis as an example of a proinflammatory effect [11,12]. A recent study also described metabolic effects of G-CSF therapy in healthy stem cell donors, and these effects may influence immunoregulation [13]. Furthermore, the systemic level of the proinflammatory acute phase stimulant interleukin-6 (IL-6) is also increased for a subset of such donors [9], but it is not known which cells are responsible for this IL-6 response [6,14]. 

Optimal selection of the healthy stem cell donor is essential for outcome after allogeneic stem cell transplantation, and among the well-characterized donor risk factors are major histocompatibility complex mismatches, female donor for male patient, donor age, and Killing Immunoglobulin-like Receptor genotype [15]. As described in a recent article, several studies have now described associations between graft compositions and outcome after ALLO-SCT [16], and the first study of individualized GVHD prophylaxis based on graft composition has already been published [17]. However, several studies have demonstrated that the immunomodulatory effects of G-CSF-induced stem cell mobilization differ between healthy donors [18]. Firstly, the effects of G-CSF on serum levels of a wide range of both pro- and anti-inflammatory cytokines, as well as soluble adhesion molecules and extracellular proteases, differ between healthy donors [9,19]; Secondly, the effect of G-CSF on immunoregulatory metabolites also varies [13]; Thirdly, the numbers of different immunocompetent cell subsets vary between grafts derived from different donors [20]; Finally, a recent study suggests that the responsiveness of immunocompetent cells to G-CSF administration differs between healthy donors, i.e., there are qualitative differences, and not only quantitative differences, between grafts derived from different donors [21]. An important question is therefore whether the G-CSF induced immunomodulation is heterogeneous and whether such differences between donors have an impact on outcome after allotransplantation [16]. The aims of our present study were therefore to investigate whether IL-6 or other IL-6 family cytokines/receptors are influenced by G-CSF therapy and thereby contribute to the heterogeneity of healthy allogeneic stem cell donors, to examine whether this heterogeneity is important for outcome after allogeneic stem cell transplantation, and to elucidate whether G-CSF will alter the release of IL-6 by in vitro cultured monocytes and/or fibroblasts. 

IL-6 depends on gp130 for transmembrane signaling, and C-reactive protein (CRP) production is mainly driven by classical IL-6 signaling (dependent on membrane-bound IL-6 receptors) whereas trans-signaling (dependent on soluble IL-6 receptors) seems less important [22]. G-CSF increases IL-6 levels and would therefore be expected to increase the acute phase reaction (including CRP). However, one should emphasize that the final effect of G-CSF on CRP levels depends on the biological context and G-CSF can reduce the acute phase responses after tissue injury [23]. Other cytokines that depend on gp130 for signal transduction (e.g., other IL-6 family members) may then induce an acute phase response in the absence of IL-6 [24,25]. Taken together, these observations suggest that the balance between pro- and anti-inflammatory effects of G-CSF and IL-6 depends on the clinical context. This is also supported by previous studies of post-transplant G-CSF therapy in allotransplant recipients; whether G-CSF therapy will influence post-transplant survival depends on the conditioning therapy and the type of stem cell graft [26]. We have previously reviewed the scientific evidence for a role of IL-6 in the development of immune-mediated complications after allotransplantation [27], and previous studies have also shown that IL-6 serum levels are altered during G-CSF mobilization for a large subsets of healthy stem cell donors [9,19,28]. Even though risk-adapted GVHD prophylaxis based on variations in graft composition is already considered, a better understanding of the mechanisms behind, and the consequences of, donor and graft heterogeneity is needed, including the possible roles of the IL-6 family and the contribution of G-CSF to the heterogeneity. In our present study; we therefore investigated effects of G-CSF on systemic levels of CRP and IL-6 cytokine family members in healthy stem cell donors.

## 2. Results

### 2.1. Healthy Stem Cell Donors Are Heterogeneous with Regard to Ongoing Acute Phase Reaction and the G-CSF Therapy Causes a Further Increase of CRP Levels for a Subset of Donors

**Figure 1:**
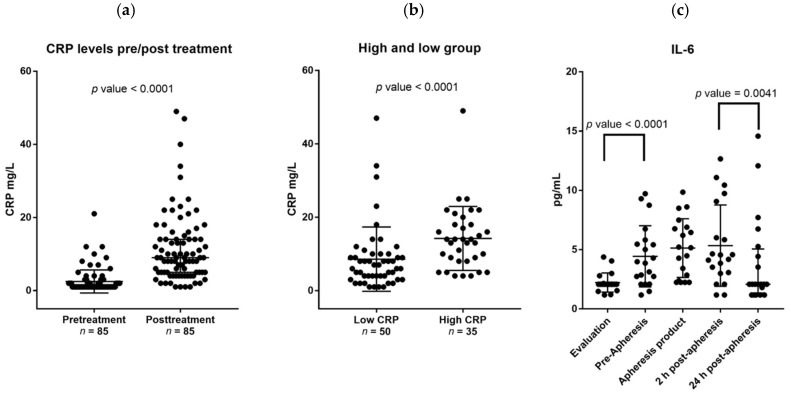
Effects of granulocyte colony-stimulating factor (G-CSF) on C-reactive protein (CRP) and systemic interleukin-6 (IL-6) levels. All results are presented as the levels for individual patients, the median levels and the 75% percentiles. (**a**) This figures shows CRP level prior to (pretreatment) and after four days of G-CSF administration (post-treatment) for all donors with detectable CRP level at these two time points. A significant increase in CRP levels was observed after G-CSF treatment; (**b**) The figure shows a comparison between the differences in CRP levels (i.e., levels during G-CSF minus the pretherapy level; mg/L) for those patients who had low (≤2 mg/L) and high pretherapy CRP level (>2 mg/L); (**c**) This figure presents the variations in serum IL-6 levels (pg/mL) for 20 healthy stem cell donors during mobilization and harvesting of peripheral blood stem cells; each dot represents the observations for one patient at the given time point. Treatment with G-CSF induced a significant increase in systemic IL-6 levels (evaluation versus pre-apheresis levels, *p*-value < 0.0001). This increase was maintained 2 h after apheresis, i.e., the pre-apheresis levels did not differ significantly from 2 h postapheresis levels (*p*-value 0.275). However, the IL-6 levels decreased significantly from 2 h to 24 h postapheresis (2 h postapheresis levels versus 24 h postapheresis levels, *p*-value <0.0041), and this decrease represents a normalization of the systemic IL6 levels during the first 24 h after apheresis (i.e., 24 h postapheresis levels versus pretherapy/evaluation levels, *p*-value 0.123). The median time from donor evaluation (the first sample, also referred to as the pretreatment sample) to start of G-CSF therapy was 16 days. The levels in the graft supernatants (apheresis product, 19 patients tested) are also presented.

Data were available for 39 female and 59 male donors; the clinical characteristics of the recipients and their matched family donors are given in Material and Methods, Section 4.1. The median number of circulating CD34^+^ cells on the day of stem cell collection was 51.2 × 10^6^/L (range 15.3–160.7). Age was the only factor associated with reduced level of circulating CD34^+^ cells (Spearman’s rho −0.420 *p* < 0.01). Their serum CRP levels were generally low with 75% having CRP level <2 mg/L and 50% below the lower limit of detection (1 mg/L). However, CRP levels were significantly higher (median increase 7 mg/L; median level 9.5 mg/L with range 1 to 49 mg/L, *p* < 0.01) after four days of G-CSF therapy. Those patients with relatively high pretherapy CRP level (i.e., >2 mg/L) also had significantly higher CRP level than the others during G-CSF therapy (Figure 1a). 

The donor heterogeneity with regard to serum CRP levels was maintained during G-CSF therapy (Figure 1b). Furthermore, donors in the fourth age quartile had significantly higher CRP level than the younger donors both prior to G-CSF (median 2 mg/L with range 3 to 21 mg/L versus median 1 mg/L with range 2 to 12 mg/L) and during G-CSF treatment (median 13.3 mg/L with range 1 to 49 versus median 8 mg/L with range 1 to 47 mg/L). Although both age and pretherapy CRP level were associated with higher CRP levels during G-CSF therapy in univariate analyses, age was not significant when corrected for pretreatment CRP levels (Table 1).

Only pretherapy CRP levels (but not CRP levels during the G-CSF therapy) showed a weak but significant correlation with the levels of circulating CD34^+^ cells after four days with G-CSF therapy (Spearman’s rho −0.21, *p*-value < 0.03). Finally, the donor CRP levels before and during G-CSF therapy were not associated with risk of acute GVHD or overall survival of the stem cell recipients. 

### 2.2. G-CSF Therapy of Healthy Stem Cell Donors Is Associated with Increased Serum Levels of IL-6 Whereas the Levels of Other IL-6 Family Members Are Not Altered during Stem Cell Mobilization

We investigated the IL-6 cytokine family in more detail for an unselected subset of 20 healthy donors (11 women, nine men). Serum samples were then collected before and during (i.e., immediately before apheresis) G-CSF therapy, immediately after and 24 h after apheresis. Graft supernatants were also analyzed. The levels of IL-6 family members were determined for all samples (Figure 1c). Low IL-6 serum levels were detected in pretherapy samples for all donors, the levels increased significantly during G-CSF treatment (*n* = 20, *p* < 0.001) and were even higher in graft supernatants. However, IL-6 levels normalized within 24 h after apheresis (i.e., 26–30 h after the last G-CSF injection). 

As can be seen from Table 2, the sIL-6R levels were not altered by the G-CSF therapy, but the sIL-6R levels were significantly increased in the graft supernatants and in the serum 24 h after stem cell harvesting. Furthermore, the levels of ciliary neutrophilic factor (CNTF), oncostatin M (OSM), and IL-31 showed no variations during stem cell mobilization and collection, but for OSM and IL-31 significantly increased levels were detected in the stem cell grafts compared with the serum levels (Table 2). Finally, leukemia inhibitory factor (LIF) could not be detected in any samples for the 10 patients examined.

Graft levels were significantly higher than the postapheresis peripheral blood levels especially for IL-31 and OSM, whereas the differences between graft and serum levels for sIL-6R and CNTF reached only borderline significance (Table 2). The ratio between serum levels of sIL-6 receptor and sgp130 is termed the IL-6 buffer; this ratio was not altered by G-CSF therapy.

### 2.3. CRP Levels during G-CSF Therapy Are Significantly Correlated with the Oncostatin M Serum Levels but There Is No Association with the Corresponding Serum IL-6 Levels

The systemic IL-6 and CRP levels showed a significant correlation before G-CSF therapy (Spearman’s rho 0.51, *p*-value = 0.02), but this correlation was absent during G-CSF treatment (Spearman’s rho 0.05, *p*-value 0.86) when the CRP levels showed a significant correlation with serum OSM levels (Spearman’s rho 0.521, *p*-value 0.022). Finally, age showed a significant association with peripheral blood CD34^+^ cell level at the time of harvesting, but the CD34^+^ cell levels did not show significant associations with the levels of any IL-6 family cytokines/receptors at any of the investigated time points. 

### 2.4. Systemic (Serum) Levels of IL-6 Family Cytokines and Especially the Oncostatin M Levels Vary between Donors Both When Tested before and during G-CSF Therapy 

**Figure 2:**
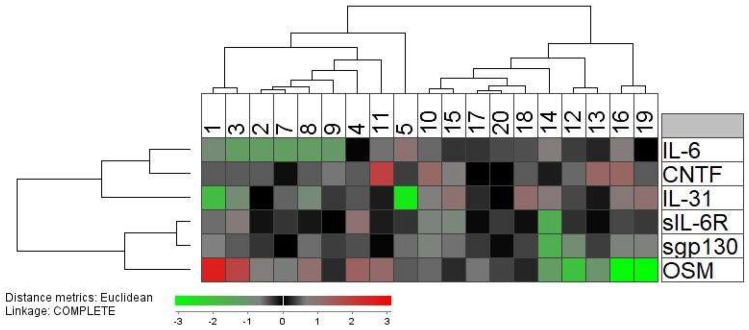
An unsupervised hierarchical clustering analysis of the graft supernatant levels of IL-6 family cytokines/receptors after stem cell mobilization by G-CSF and harvesting by leukapheresis. The analysis included 19 donors because a graft sample was not available for patient 6. The mediator concentrations were normalized to the corresponding median level for each mediator and, thereafter, log2 transformed before an unsupervised hierarchical clustering with Euclidian distance measurement and complete linkage was performed. The color scale thus corresponds to the Euclidian distance from the median since values were normalized to the corresponding median value, i.e., two measurements with the same color show the same distance from the median. The results are presented as dendrograms and a heat map for visualization and interpretation. The individual donors are indicated at the top of the figure whereas the different mediators are presented vertically in the right part of the figure.

Even though IL-6 was the only cytokine that was significantly altered during G-CSF therapy and apheresis, it can be seen from Table 2 that the other IL-6 family cytokines, and especially OSM, showed a considerable variation among donors. Therefore, we did an unsupervised hierarchical clustering analysis of the graft levels immediately after apheresis to further characterize and visualize the overall influence of mobilization and harvesting (Figure 2; 19 patients included, graft levels were not available for patient 6). This analysis identified two main patient clusters; the left cluster included patients that generally showed relatively high levels of OSM and low IL-6 levels, whereas many of the patients in the right cluster showed low OSM levels and higher IL-6 levels. The two clusters did not differ with regard to patient age or gender distribution.

### 2.5. The Levels of Immunocompetent Cell Subsets in Peripheral Blood and Allogeneic Stem Cell Grafts Vary between Healthy Donors: Studies of Associations between Serum Levels of IL6 Family Cytokines, Circulating Immunocompetent Cells, and Graft Content of Immunocompetent Cells

We investigated the graft composition and the peripheral blood levels of total T cells, CD4^+^ T cells, CD8^+^ T cell, B cells, monocytes, and natural killer (NK) cells together with the levels of CD34^+^ cells for our healthy stem cell donors (Table 3). The peripheral blood levels were determined after four days of G-CSF treatment immediately before stem cell harvesting by leukapheresis. There was a considerable variation between the donors with regard to the peripheral blood levels of all immunocompetent cell subsets; the largest variation being observed for CD16^+^ NK cells. The number of harvested graft cells on the first day of apheresis (i.e., after four days of G-CSF treatment) also varied considerably, especially for NK cells, but also for B cells and monocytes. 

We investigated the associations between the levels of circulating immunocompetent cells and the systemic (serum) levels of each individual IL-6 family cytokine or CRP (Table 3, upper part). The most striking observations were the inverse correlations between serum sgp130 and the levels of circulating total T cells, CD4^+^ and CD8^+^ T cells, B cells, and CD34^+^ cells; an additional inverse correlation was observed between sIL-6R and circulating CD4^+^ T cells. Finally, total T cell levels in the blood were also correlated with the CNTF levels. These observations suggest that IL-6 family mediators, and especially gp130/IL-6R, are involved in the trafficking/mobilization of immunocompetent cells during G-CSF therapy of healthy donors. 

We also investigated associations between the amounts of harvested immunocompetent cells and serum levels of CRP and IL-6 family cytokines (i.e., graft composition on the first day of apheresis). The graft composition will then reflect an overall effect of G-CSF therapy and the leukapheresis procedure. CRP levels then showed significant inverse correlations with the graft numbers of CD8^+^ T cells and B cells whereas IL6 was significantly associated with levels of B cell in the graft (Table 3, lower part). 

We compared the peripheral blood and graft levels of the various immunocompetent cell subsets for the donor subsets identified in the clustering analysis presented in Figure 2; i.e., whether the levels of immunocompetent cells were dependent on variations in the overall IL-6 family profile. The left cluster showed a lower level of total B cells (Mann–Whitney *U* test; *p* = 0.03); this was the only significant difference that was detected. Finally, the donor age did not show any significant associations with graft or peripheral blood levels of immunocompetent cells. 

### 2.6. G-CSF Can Modulate IL-6 Release by Immunocompetent and Mesenchymal Cells

IL-6 is released by immunocompetent cells and various stromal cells during acute infections in response to danger-associated or pathogen-associated molecular patterns recognized by Toll-like receptors (TLRs) [27,29]. However, a wide range of other endogenous molecules have also been identified as TLR ligands that are able to induce TLR-initiated intracellular signaling, and these observations may suggest that TLRs are important, not only during infections or inflammation, but possibly also for the normal immunological surveillance or homeostasis [30]. Various TLRs are differentially expressed by monocytes, fibroblasts, and mesenchymal stem cells (MSCs) [31], and TLR ligation may therefore influence their functional status in vivo. For these reasons we investigated whether G-CSF can modulate the in vitro release of IL-6 by monocytes, fibroblasts, or mesenchymal stem cells in the presence of various TLR-ligands.

**Figure 3:**
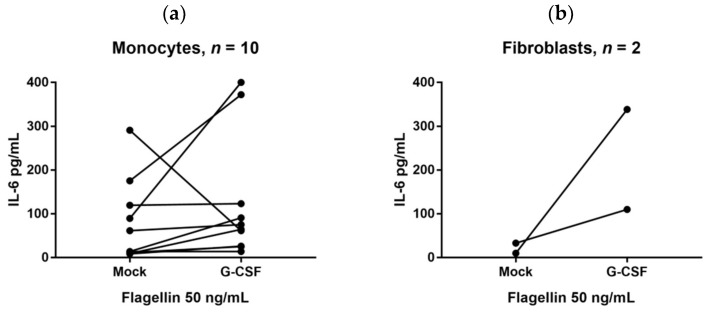
IL-6 release by monocytes derived from 10 healthy individuals (**a**). The cells were cultured with and without exogenous G-CSF 50 ng/mL in the presence of the TLR agonists Flagellin (TLR5) 50 ng/mL. The results are presented as the IL-6 levels in culture supernatants; (**b**) IL-6 release by HFL1 and Hs27 fibroblasts cultured with and without exogenous G-CSF 50 ng/mL in the presence of Flagellin (TLR5) 50 ng/mL; the results from a typical experiment are presented. The results are presented as the IL-6 levels in culture supernatants; the effect of G-CSF on IL-6 release by fibroblasts was detected in six independent experiments.

We investigated the effects of G-CSF on the IL-6 release by monocytes in the presence the TLR agonists Pam3CSK4 (TLR1/2), LPS (TLR4) or Flagellin (TLR5), R837 (TLR7 > TLR8), and R848 (TLR7/8). Based on initial dose–response experiments we investigated the G-CSF effects in the presence of two different concentrations for each agonist, both concentrations being lower than the concentrations needed for induction of maximal IL-6 release. These results are summarized in Table 4. Monocytes derived from 10 healthy individuals were investigated. Firstly, the IL-6 release by normal monocytes showed a wide variation between the healthy individuals for all agonists investigated. Secondly, we defined a strong/significant G-CSF effect as at least a twofold alteration. For all agonists a strong G-CSF effect was only observed for a subset of healthy cell donors, i.e., the G-CSF effect differed between individuals, and a strong effect was most common in the presence of the TLR5 agonist Flagellin. The Flagellin 50 ng/mL results are presented in Figure 3a. Finally, the overall results presented in Table 4 showed that G-CSF usually increased the IL-6 levels, but for certain donor/agonist combinations decreased levels were seen.

We also examined the effect of exogenous G-CSF on IL-6 release by two fibroblast cell lines derived from different individuals and tissues and by MSCs derived from a healthy individual. The G-CSF effect was tested in the presence of three TLR agonists: Pam3CSK4 1 ng/mL (TLR1/2 agonist), LPS 5 ng/mL (TLR4 agonist), and Flagellin 10 ng/mL (TLR5 agonist). Both fibroblast cell lines showed increased IL-6 release in the presence of G-CSF. Increased IL-6 release by fibroblasts release was demonstrated in six independent experiments; it was detected early during culture as well as later when cells were close to confluence, and a strong effect was especially seen in the presence of Flagellin (Figure 3b). Finally, enriched MSCs from a healthy donor showed constitutive IL-6 release that was increased in the presence of exogenous G-CSF. 

Taken together these results suggest that both immunocompetent and stromal cells contribute to the G-CSF induced IL-6 response in healthy individuals, but their contribution possibly differs between various individuals and also seems to depend on the microenvironment of the cells as illustrated by the different G-CSF effects in the presence of various TLR agonists.

## 3. Discussion

Previous studies suggest that healthy stem cell donors are heterogeneous with regard to the effects of G-CSF on donor immunoregulation and the number, as well as the functional status, of immunocompetent graft cells [9,13,15,18,19,20,21]. One of these studies even suggests that G-CSF induced donor heterogeneity is important for outcome after allotransplantation [21]. We have previously reviewed and discussed the available evidence for a role of IL-6 in the development of immune-mediated complications after allotransplantation [27]. Previous studies have also shown that systemic IL-6 levels in healthy stem cell donors can be altered by G-CSF therapy; these effects are divergent, and although increased levels are seen for most donors, a minority of them show decreased systemic IL-6 levels in response to G-CSF [9,19,28]. In our present study we observed that healthy donors undergoing G-CSF induced stem cell mobilization and harvesting by leukapheresis are heterogeneous, both with regard to the G-CSF induced acute phase reaction and effects of G-CSF on systemic levels of various IL-6 cytokine/receptor family members.

Several recent studies have described associations between graft composition and post-transplant outcome, e.g., high CD8^+^ graft cells associated with decreased relapse risk [32] and increased regulatory T cells associated with decreased nonrelapse mortality [33]. The first study investigating individualized risk-adapted prophylaxis against immune-mediated complications based on graft composition has already been published [17]. However, a better understanding of the molecular mechanisms behind, and the consequences for, the recipients of differences in graft composition is needed as a scientific basis for further studies of possible interventions, e.g., in vivo graft manipulation, ex vivo graft manipulation, risk-adapted individualized prophylaxis, or early therapeutic intervention based on biomarker evaluation before clinical signs of complications [16]. 

Previous studies suggest that G-CSF-induced stem cell mobilization in healthy individuals has a clinically negligible effect on CRP levels with most donors still having CRP levels below 2 mg/L after G-CSF administration [34,35]. In contrast, we observed an increase of at least 9.5 mg/L for a large subset of donors, especially elderly donors. The only other factor predicting this CRP increase was the pretreatment CRP levels, implicating that signs of pretreatment inflammation potentiates the effects of G-CSF on the acute phase reaction. 

IL-6 and CRP levels are usually highly correlated [36]; this was also seen for the pretreatment levels for our stem cell donors. However, we did not detect any significant association between CRP and IL-6 levels during G-CSF treatment, but CRP levels were significantly correlated with OSM levels even though the OSM levels did not increase in response to G-CSF. G-CSF itself is not able to induce CRP production in hepatocytes [37]. Taken together, these observations suggest that G-CSF induced CRP release is independent of the IL-6 response and rather caused by a G-CSF induced modulation of OSM effects. Even though tumor necrosis factor alpha (TNF-α) or IL-1 can induce CRP release [38], these two cytokines are less likely to contribute because G-CSF decreases their systemic levels [8]. Finally, the ratio between serum levels of sIL-6 receptor and sgp130 is termed the IL-6 buffer; this buffer regulates the proinflammatory effects of IL-6, including its effects on the acute phase response/CRP levels [39,40]. However, the IL-6 buffer was not altered during G-CSF therapy and therefore is unlikely to be responsible for the increased CRP levels during G-CSF therapy [24,25]. 

We observed an association between the G-CSF induced acute phase reaction and OSM levels. OSM is released by various immunocompetent cells; it can initiate acute phase reactions and is also involved in tissue repair [41,42]. The OSM receptor uses gp130 as the signaling subunit of the receptor complex; this is similar to the other IL-6 family cytokines, but OSM can also utilize the LIF receptors for signal transduction [27]. OSM seems to have the broadest downstream signaling profile among the IL-6 family members and activates Janus kinase (Jak)/ Signal transducer and activator of transcription (STAT) signaling, the extracellular signal–regulated kinases (ERK) and c-Jun N-terminal kinase, phosphatidyl-inositole-3-kinase/ Protein Kinase B (Akt) signaling, as well as protein kinase C delta [41,43]. 

OSM is also regarded as a disruptor of epithelial barrier functions, it is a biomarker for active inflammation in rheumatoid arthritis and increased levels are also reported in allergic rhinitis, psoriasis, and asthma [42]. It seems to have a very complex role in the regulation of inflammation by enhancing the maturation of dendritic cells and thereby increasing their IL-12 release, increasing T cell proliferation, and increasing the release of Interferon-γ [44]. However, it also seems to skew monocyte differentiation into the anti-inflammatory M2 phenotype and does not stimulate development of dendritic cells from monocytes. In vivo studies suggest that OSM has anti-inflammatory effects mediated by inhibition of IL-1 and TNF-α responses, and it seems to suppress inflammation in animal models of autoimmune diseases [45]. OSM does not seem to have direct effects on Th17 cells and regulatory T cells [44]. Taken together, these observations suggest that the predominant effects of OSM depend on the biological context. Our present results suggest that its proinflammatory effects (i.e., the effects on the acute phase reaction) vary between, and thereby contributes to, the heterogeneity of healthy stem cell donors (Table 2, Figure 2), and this variation during G-CSF therapy and in graft supernatants suggests that OSM can alter the functional status of at least certain subsets of graft immunocompetent cells. Even though the possible role of OSM in allotransplant recipients has not been addressed previously, our knowledge about OSM from other studies suggests that it may contribute to the post-transplant outcome (e.g., development of immune-mediated toxicity) in allotransplant recipients through the acute phase reaction, immunoregulatory and proinflammatory effects, modulation of inflammatory resolution and tissue repair after inflammation, or effects on epithelial barrier functions. 

The peripheral blood levels and the corresponding graft amounts of immunocompetent cells showed a wide variation between healthy donors (Table 3), and the widest variation in peripheral blood levels was seen for NK cells. The NK cells seem important for outcome after stem cell transplantation [46]. Previous studies have also demonstrated that NK cells show a transient functional alteration following G-CSF mobilization with decreased proliferative capacity; this effect also varies between patients [47,48]. Thus, healthy stem cell donors show both a quantitative and qualitative NK cell heterogeneity after G-CSF mobilization. 

The levels of several circulating immunocompetent cell subsets showed an association with the systemic levels of sgp130 that serves as an important modulator of IL-6 signaling through its binding to soluble IL-6R [27]. This observation suggests that IL-6 family cytokines, and especially IL-6, are important for immunocompetent cell mobilization and may contribute to the donor heterogeneity observed during G-CSF therapy. These associations were not detected for the allografts, probably because graft levels also depend on factors related to the apheresis and graft preparation and not only on the G-CSF mobilization [28]. 

We also investigated whether G-CSF could increase IL-6 release by in vitro cultured cells. IL-6 can be released by several immunocompetent as well as mesenchymal cells [27], and in our present study we included only monocytes together with fibroblasts and normal mesenchymal stem cells. We then used an in vitro model where monocytes and mesenchymal cells were cultured in the presence of TLR agonists; in our opinion this is a more physiological model than culture in medium alone because a wide range of endogenous TLR ligands have now been identified and are expected to be present in the in vivo microenvironments of these cells [49]. A strong/significant alteration of the IL-6 release in the presence of G-CSF was defined as a two-fold alteration. It can be seen from Figure 3 that the in vitro G-CSF effects on the monocyte release of IL-6 differed between healthy individuals (although increased IL-6 levels were most common). Previous in vivo studies also suggest that the effects of G-CSF on IL-6 vary between individuals, i.e., the effect of G-CSF therapy on systemic IL-6 levels of healthy stem cell donors differs and both increased, unaltered, and decreased systemic levels can be seen [9,19].

We investigated monocyte/fibroblast/mesenchymal stem cell release of IL-6 in an experimental model based on serum-free (i.e., possibly suboptimal) culture medium; this was use to minimize the risk of having TLR ligands in the medium. Our model is thus based on the presence of one ligand, whereas we would expect several endogenous TLR ligands to be present during physiological conditions. For these reasons we would emphasize that these results should be interpreted with great care and additional studies in other experimental models are needed to characterize, in greater detail, the effect of G-CSF IL-6 release by such cells.

Fibroblasts express a wide range of TLRs, and we also observed increased IL-6 release for both fibroblast cell lines in the presence of various TLR agonists. The constitutive IL-6 release by MSC was also increased by G-CSF. Taken together these observations suggest that various cells contribute to the IL-6 response during G-CSF therapy. This is similar to the IL-6/CRP responses during infections where both immunocompetent and mesenchymal cells contribute to these responses [49]. 

Several observations suggest that immunoregulatory events early after stem cell transplantation are important for the outcome after ALLO-SCT, especially the risk of GVHD, for example, the need for early initiation of GVHD prophylaxis and the association between pretransplant conditioning, post-transplant G-CSF therapy, and risk of post-transplant outcome [26]. Furthermore, IL-6 seems important in the development of immune-mediated complications after ALLO-SCT and is regarded a possible therapeutic target in GVHD [27]. However, only future clinical studies can clarify whether G-CSF induced donor heterogeneity, including differences in acute phase reactions and IL-6 family cytokine levels, has any impact on the outcome for the allotransplant recipients. 

## 4. Material and Methods

### 4.1. Patient Studies and Donor Samples

All studies were approved by the Regional Ethics Committee III, University of Bergen, Norway (REK VEST 2013/634 30 April 2013 and REK VEST 2015/1410, 02 July 2015). Only matched related donors (median age 49 years, range 18–77 years) mobilized with G-CSF 5 μg/kg twice daily were included. The donor and patient characteristics are given in Table 5. These recipients/donors represent an unselected cohort. The routine GVHD prophylaxis was ciclosporin A plus methotrexate. All donors were selected according to the generally accepted suitability criteria [50]. They were all healthy and without any signs of intercurrent disease at the times of evaluation, G-CSF therapy, and stem cell harvesting. Unless otherwise stated samples were collected between 8:00 am and 11:00 am in the morning. Twenty unselected donors were included in the cytokine studies (median age 51 years, range 25–73 years).

Stem cell collection was commenced after four days of G-CSF if the number of circulation CD34^+^ cells was sufficient. Samples were collected before and after 4 days of G-CSF therapy, immediately after leukapheresis, and approximately 24 h after start of leukapheresis. Graft supernatants were also collected. Samples were centrifuged at 1310× *g*, transferred onto cryotubes within 2 h after sampling, and stored at −70 °C until analyzed. Bio-Plex kits were used to analyze the levels of soluble mediators (Bio-Rad, Hercules, CA, USA), using the Luminex^®^200™ Bio-Rad platform. CRP was analyzed immediately after sampling by an immunoturbidimetric method (Roche; Basel, Switzerland); the lower detection limit being 1 mg/L.

### 4.2. Flow Cytometric Analysis

Peripheral blood and graft levels of immunocompetent cells were analyzed by flow cytometry. Briefly, peripheral blood mononuclear cells and graft cells were cryopreserved in DMSO and stored in liquid nitrogen until analyzed [21,51]. The cells were thawed and the near-IR fluorescent reactive dye (LIVE/DEAD Fixable Dead Cell Stain Kits, Molecular Probes, Eugene, OR, USA) was used for identification of viable cells. Cells were thereafter stained with CD3-PE-Cy7 (SK7), CD4-PerCP-Cy5.5 (RPA-T4), CD8-V500 (RPA-T8), CD16-Ax647 (3G8), CD19-PerCP-Cy5.5 (SJ25C1), and CD56-PE (B159) (all from Becton Dickinson Biosciences; BD Pharmingen, San Diego, CA, USA). We determined the numbers of CD3^+^ T cells, CD4^+^ and CD8^+^ T cell subsets, B cells (CD19^+^), and NK cells (CD16^+^CD56^+^) by using a FACS Canto II flow cytometer (Becton Dickinson Biosciences-Immunocytometry Systems; San Jose, CA, USA). The data were analyzed using FlowJo software version 10.2 (FlowJo LLC, Ashland, OR, USA). The monocyte levels were determined by multi-angle polarized scatter separation (MAPSS) optical flow cytometry (Cell-Dyn Sapphire analyzer; Abbot Diagnostics, Santa Clara, CA, USA).

### 4.3. In Vitro Culture of Monocytes and Fibroblasts

Samples were collected from healthy blood donors at Haukeland University Hospital. Monocytes from healthy donors were isolated from gradient-separated peripheral blood mononuclear cells (PBMCs) by negative selection using the human Monocyte Isolation Kit II (Miltenyi; Bergisch Gladbach, Germany). The isolation was performed according to the manufacturer’s instructions. Flow cytometric analysis verified that the purity was ≥95%. The Hs27 skin fibroblasts (ATCC CRL1634; Manassas, VA, USA) and HFL1 fetal lung fibroblasts (ATCC CRL153) were also examined together with mesenchymal stem cells (MSC) derived from a healthy individual (Cambrex BioScience; Walkersville, MD, USA). 

Cells were cultured with each of the TLR agonists Pam3CSK4 (TLR1/2 agonist; tested at 1 and 5 ng/mL), lipopolysaccharide (LPS) (TLR4 agonist; 0.1, 0.5, 5, and 10 ng/mL), Flagellin (TLR5 agonist; 10 and 50 ng/mL), R837 (TLR7 > TLR8 agonist; 0.5 and 1.0 mg/mL), and R848 (TLR7/8 agonist; 50 and 100 ng/mL) (Invitrogen; San Diego, CA, USA), with and without G-CSF 50 ng/mL (Peprotech; Rocky Hill, NJ, USA). Monocytes (50,000 cells/mL, 1 mL/well; Multiwell™ 48 well culture plates, Falcon, Franklin, NJ, USA) were incubated in RPMI 1640 (Sigma-Aldrich; St. Louis, MO, USA) with TLR-agonists ± G-CSF for 24 h before harvesting of supernatants. Fibroblasts (10,000 cells/mL, 2 mL/well; Nunclon Delta Surface Thermofisher 6-well culture plates; Roskilde, Denmark) were incubated in Dulbecco’s Modified Eagle’s Medium (Sigma) for 24 h before TLR agonists/G-CSF were added and supernatants harvested 24 h later. MSCs (5000 cells/mL, 2 mL/well; Nunclon Delta Thermo-Fischer 6-well culture plates) were also incubated for 24 h in mesenchymal stem cell medium alone (MSCGM™; Lonza; Basel, Switzerland) for 24 h before TLR-agonists/G-CSF were added and supernatants harvested 24 h later. Cultures were incubated at 37 °C in a humidified atmosphere of 5% CO2. Supernatants were stored at −80 °C until IL-6 analysis (Quantikine ELISA kits; R&D Systems Minneapolis, MN, USA). These mediator analyses were performed in duplicates, and the variation between duplicates was generally less than 10%. 

### 4.4. Statistical Analyses

Statistical analyses of clinical variables were performed using Stata Version 14 (StataCorp. 2009; Stata Statistical Software, College Station, TX, USA) and Graphpad Prism (GraphPad Software, Inc., La Jolla, CA, USA). Differences were regarded as statistically significant when *p*-values < 0.05.

## Figures and Tables

**Table 1:** A summary of the linear regression model of the effects of pre-G-CSF CRP levels and age on CRP levels after granulocyte colony-stimulating factor (G-GSF) administration. Age was initially entered as three different dummy variables corresponding to the second, third, and fourth quartile. Only age above or below 57 years had a significant effect on CRP levels in univariate analysis.

Covariate	Univariate			Multivariate		
	Coefficient	SE ^1^	*p*-Value	Coefficient	SE ^1^	*p*-Value
Pre G-CSF CRP level	1.48	0.31	<0.01	1.40	0.32	<0.01
Age ^2^	5.30	2.45	0.03	2.16	2.39	0.37

^1^ Standard error of the mean; ^2^ Age below or above 57 years of age.

**Table 2:** Serum levels of IL-6 family cytokines at four different time points during stem cell mobilization and harvesting; the levels in graft supernatants are also included as a comparison. The results for 20 healthy stem cell donors (median age 51 years, range 25–73 years) are summarized, and all the results are presented as the median level and the variation range. All concentrations are given as pg/mL, and statistically significant alterations compared with the pretherapy levels (before G-CSF therapy) are marked in bold (Mann–Whitney *U* test). Graft levels were only available for 19 patients, and statistically significant differences between graft levels and postapheresis levels are indicated in the table (* *p* < 0.05, ** *p* < 0.01).

Mediator	Before G-CSF	During G-CSF (Pre-apheresis)	Graft Supernatant	2 h after Apheresis	24 h after Apheresis
IL-6	2.1	**3.9**	**5.2**	**4.4**	2.1
	(1.2–4.4)	**(1.2–9.7)**	**(2.2–9.9)**	**(1.2–12.7)**	(1.2–14.2)
sgp130	19,197	17,239	22,985	17,429	18,914
	(86–26,942)	(7004–28,049)	(7666–36,063)	(9723–40,714)	(10,596–32,561)
sIL-6R	4400	3952	**6101**	4401 *	4692
	(26–6189)	(1932–7938)	**(2103–11,681)**	(2181–11,843)	(2252–12,936)
IL-31	6.7	6.4	**37.8**	5.3 **	6.7 **
	(3.6–21.8)	(3.6–19.5)	**(5.8–76.8)**	(3.6–15.3)	(3.6–9.8)
OSM	29	31	**94**	32 **	36 **
	(7–214)	(8–229)	**(11–538)**	(8–137)	(10–214)
CNTF	624	571	677	649	571 *
	(470–1543)	(470–2019)	(494–2507)	(470–1892)	(470–1710)

**Table 3:** Serum levels of soluble mediators and their associations with levels of immunocompetent and CD34^+^ cells in peripheral blood and stem cell grafts. We investigated the levels of six different immunocompetent cell subsets for 20 healthy stem cell donors. Serum levels and levels of circulating cells were determined after four days of G-CSF therapy before apheresis; graft composition was analyzed for the leukapheresis on day 4. For immunocompetent cells the results are presented as the cell number × 10^9^/L in peripheral blood/grafts; for CD34^+^ cells the levels are presented as the number × 10^3^/mL in peripheral blood and × 10^9^/L in the grafts. Correlation coefficients (Spearman’s rho) between serum levels of IL-6 family cytokines/receptors/CRP and immunocompetent cell subsets in the graft and peripheral blood are also presented. Significant correlations are highlighted in bold (* *p*-value between 0.05 and 0.01, ** *p*-value below 0.01).

**The Peripheral Blood Levels of Immunocompetent Cell Subsets**								
**Leukocyte subset**	**Peripheral Blood Level ^1^**	**IL-6**	**sIL-6R**	**sgp130**	**IL-31**	**OSM**	**CNTF**	**CRP**
T cells, total (CD3^+^)	3.31 (1.29–4.17)	−0.042	−0.508	−0.697 **	0.244	−0.511	0.654 *	0.156
CD4^+^ T cells	2.54 (0.92–3.47)	0.046	−0.582 *	−0.609 *	0.354	−0.495	0.427	0.229
CD8^+^ T cells	0.60 (0.24–1.08)	−0.135	−0.205	−0.557 *	0.104	−0.275	0.555 *	0.097
B cells (CD19^+^)	0.41 (0.21–1.77)	0.289	−0.310	−0.719 **	0.525	−0.423	0.507	0.384
NK-cells (CD3^−^ CD56^+^)	0.30 (0.07–0.77)	−0.449	0.165	0.181	−0.020	0.366	−0.074	−0.249
Total monocytes	2.4 (0.90–3.9)	−0.065	−0.164	−0.296	0.276	−0.046	0.362	0.210
CD34^+^ cells	40.2 (16.7–148)	−0.21	−0.32	−0.54 *	0.47	0.37	0.33	0.045
**The Graft Composition of Immunocompetent Cell Subsets**								
**Leukocyte subset**	**Graft Level ^1^**	**IL-6**	**sIL-6R**	**sgp130**	**IL-31**	**OSM**	**CNTF**	**CRP**
T cells, total (CD3^+^)	22.78 (8.41–42.81)	−0.088	0.328	0.294	0.097	−0.074	−0.358	−0.539 *
CD4^+^ T cells	17.55 (6.02–31.66)	0.073	0.459	0.516	−0.162	−0.196	−0.176	−0.444
CD8^+^ T cells	4.64 (1.30–9.74)	−0.068	0.336	0.204	0.087	−0.007	0.268	−0.592 *
B cells (CD19^+^)	3.63 (0.00–12.76)	0.534 *	0.363	0.169	0.184	0.385	0.277	−0.622 *
NK-cells (CD3^−^ CD56^+^)	1.79 (0.40–5.50)	−0.121	0.253	0.433	0.315	0.415	0.121	−0.407
Total monocytes	12.91 (1.93–25.23)	−0.248	0.071	0.100	0.248	0.324	−0.054	−0.256
CD34^+^ cells	0.43 (0.085–201)	−0.026	0.319	0.125	−0.258	−0.088	0.400	−0.009

^1^ Peripheral blood levels: The vertical column presents the cell subset, the horizontal line the serum soluble mediator.

**Table 4:** The effect of G-CSF on IL-6 release by monocytes derived from 10 healthy individuals. Enriched monocytes were incubated with various TLR agonists (for each individual two different concentrations were tested), and the IL-6 supernatant levels were compared for cultures with G-CSF and corresponding control cultures without G-CSF. The table presents the median and range of the IL-6 concentrations for all 20 cultures with each agonist (i.e., 10 healthy individuals tested with two concentrations of each agonist); control cultures of monocytes incubated in medium alone showed undetectable IL-6 levels. A significant difference was defined as at least a twofold increase/decrease in the presence of G-CSF—at least 20 pg/mL. Divergent effects between the two concentrations of an agonist were not observed for any agonist/donor combination. The dark color indicates a significant G-CSF induced IL-6 increase for at least one of the two agonist concentrations tested, whereas the bright color indicates a significant decrease. Cultures marked with nt means that these were tested with different LPS concentrations (0.1 and 0.5 ng/mL); none of these alternative LPS/donor combinations showed any significant influence of G-CSF on the IL-6 levels. Monocytes cultured in medium alone without G-CSF/TLR agonists showed undetectable IL-6 levels.

Agonist	Agonist Concentration	IL-6 Supernatant Levels (pg/mL)	Healthy Monocyte Donors									
			1	2	3	4	5	6	7	8	9	10
PAM3CSK4 (TLR1/2)	1 and 5 ng/mL	10.8 (3.1–372)										
LPS (TLR4)	5 and 10 ng/mL	3.1 (3.1–281)				nt	nt	nt	nt	nt	nt	nt
Flagelin (TLR5)	10 and 50 ng/mL	13.9 (3.1–291)										
R848 (TLR7 > TLR8)	50 and 100 ng/mL	3.1 (3.1–180)										
R837 (TLR7/TLR8)	0.5 and 1 mg/mL	188 (3.1–395)										

**Table 5:** The characteristics of the allotransplant recipients and their donors included in the analysis.

Recipients (*n* = 85)	Characteristics
Age, median and range (Years)	47 (18–70)
Diagnosis (number)	
AML, de novo	37
AML secondary to myelodysplastic syndrome	17
Myelodysplastic syndrome, high-risk	2
Acute lymphoblastic leukemia	15
Chronic myeloid leukemia	3
Myelofibrosis/Myeloproliferative neoplasia, unspecified	6
Chronic myelomonocytic leukemia	2
Chronic lymphocytic leukemia	2
Hodgkin’s lymphoma	1
Leukemia patients not in remission at transplantation	1
aGVHD requiring high dose steroid treatment (number) ^1^	38
Conditioning regimes (number)	
Busulfan + cyclophosphamide (myeloablative condition)	66
Fludarabine + busulfan (reduced intensity conditioning)	16
Others	3
Stem cell source (number)	
Peripheral blood mobilized stem cells	85
Bone marrow grafts	0
**DONORS (*n* = 85)**	
Sibling/other family donors	78/7
Female/Male	54/31
Age; median (range)	49 (18–77)
Female donor to male recipient	19
Number of CMV positive recipients	60
CMV positive donor to CMV negative recipient	15

^1^ The criteria for receiving high-dose systemic steroid treatment were acute GVHD grade II with gastrointestinal involvement or Grade III/IV acute GVHD.

## Abbreviations

ALLO-SCT	Allogeneic stem cell transplantation
CNTF	Ciliary neutrophilic factor
gp130	Glycoprotein 130
GVHD	Graft-versus-host disease
G-CSF	Granulocyte-colony stimulating factor
IL-31	Interleukin-31
IL-6	Interleukin-6
LPS	Lipopolysaccharide
MCSs	Mesenchymal stem cells
OSM	Oncostatin M
sIL-6R	Soluble IL-6 receptor
TLR	Toll-like receptor.
